# 2 × 4.5 kW bidirectional output near-single-mode quasi-continuous wave monolithic fiber laser

**DOI:** 10.1038/s41598-023-48478-7

**Published:** 2023-12-01

**Authors:** Xinyi Ding, Lingfa Zeng, Li Wang, Hanshuo Wu, Peng Wang, Hanwei Zhang, Xiaolin Wang, Yu Ning, Fengjie Xi, Xiaojun Xu

**Affiliations:** 1 College of Advanced Interdisciplinary Studies, National University of Defense Technology, Changsha, 410073 China; 2 Nanhu Laser Laboratory, National University of Defense Technology, Changsha, 410073 China

**Keywords:** Lasers, LEDs and light sources, Fibre lasers

## Abstract

Quasi-continuous wave (QCW) laser has a very broad application in the industrial field, especially in additive manufacturing, surface treatment, laser cutting, laser cleaning, and laser drilling. Compared with the unidirectional fiber laser, the bidirectional output can be achieved two ports high power output with only one resonator, which can greatly reduce the industrial cost. However, there are few researches on QCW fiber lasers with bidirectional output. Here, we optimized and demonstrated a bidirectional output QCW laser with output power of 2 × 4.5 kW based on a double-clad ytterbium-doped fiber with a core/cladding diameter of 25/400 μm. The peak power at both ends reached 4515 W and 4694 W, respectively. The Raman suppression ratio at both ends of A and B is about 12 dB, and the beam quality factor *M*^2^ is about 1.37 and 1.42, respectively. The corresponding optical-to-optical efficiency is 79%. To the best of our knowledge, this is the highest peak power of QCW laser with near-single-mode beam quality in a bidirectional structure laser.

## Introduction

Fiber lasers have stable high-power output, excellent beam quality, good thermal management characteristics, high conversion efficiency and small volume, which make them widely used in industrial processing, healthcare, additive manufacturing, and other fields^1^. Among them, because of the more concentrated energy and better heat dissipation effect, the pulse generated by the quasi-continuous wave (QCW) fiber laser performs better in some fields. QCW laser provides a molten pool with a higher cooling rate and more directional heat flow than continuous wave (CW), which helps to obtain more stretchable samples in laser additive manufacturing^2–4^. In the surface treatment of the material, its rapid cooling and multi-directional solidification behavior can improve the corrosion resistance of the alloy without affecting the mechanical properties of the melt layer^5^. In the process of laser cutting, QCW laser can improve the working rate and reduce the formation of the heat-affected zone. For temperature-sensitive materials and composites with differences in thermal properties, this can minimize thermal damage and avoid material delamination^6–8^. In the process of laser cleaning, by adjusting the repetition frequency and duty cycle of the QCW laser, the heat penetration depth can be controlled, and the coating can be heated to the critical temperature to achieve the purpose of removing the coating layer by layer^9^. For materials with high hardness, brittleness, or/and high reflection, which are difficult to process using traditional methods, QCW laser processing guarantees good hole circularity and less melting, allowing smooth sidewalls, minimal splash, and high uniformity of high aspect ratio through holes^10–13^. In addition, in multi-material components, QCW laser welding is proving to be an effective method for joining dissimilar metals due to its high-power density but low energy input^14^.

In recent years, remarkable achievements have been made in researching high-power QCW fiber lasers^15–22^. However, the reported high-power QCW fiber lasers are difficult to achieve good beam quality. In 2015, IPG achieved a QCW laser output with a peak power of 1.5 kW and a beam quality of *M*^2^ = 1.05^21^. Subsequently, the peak power increased to 23 kW, however, with a degraded beam quality. Using the feeding fiber with a core diameter of 100 μm, the beam-parameter product (BPP) is 4.2 mm mrad, corresponding to the beam quality factor *M*^2^ = 12.33^22^. In 2023, Wang et al*.* used a 30/600 μm double-clad ytterbium-doped fiber (DCYDF) to achieve a QCW laser output with an average output power of 973 W and a peak power of 10.75 kW at a pumping repetition rate of 1 kHz, and the beam quality factor *M*^2^ is about 1.6^23^. Better beam quality means higher brightness, and in industrial processing, lighter optical focusing devices can be used to obtain more lightweight machining heads and faster processing speeds. And fiber lasers with high beam quality can gain more significant advantages in remote processing^1^. Therefore, in order to meet the industrial needs in more scenarios, QCW fiber lasers with high beam quality and peak power need to be further developed.

Currently, the research on QCW fiber lasers only uses the unidirectional output structure. The bidirectional fiber laser only needs a single resonator to achieve two laser outputs. Compared with the unidirectional output laser, the bidirectional output laser can effectively not only reduce the cost and volume size, but also offer a higher work efficiency. The bidirectional output structure is expected to achieve higher output power with a broader industrial application prospect^24–26^. There are relatively few reports about bidirectional output lasers. In 2017, a bidirectional output monolithic laser oscillator based on a specially designed gain fiber was first proposed. However, due to the incompatibility of this gain fiber with devices based on double-clad fiber, the pumping power is limited^27^. In 2018, Wang et al. first proposed a linear cavity all-fiber laser oscillator with bidirectional output, which promoted the power increase of bidirectional lasers^26^. In 2022, Zhong et al*.* achieved the 2 × 2 kW near-single-mode CW laser with bidirectional output monolithic fiber laser, and the beam quality at both ends was about *M*^2^ = 1.5^24^. In 2023, Liu et al*.* proposed a novel bidirectional output oscillating-amplifying integrated fiber laser and achieved a CW output of 2 × 2 kW with beam qualities of about *M*^2^ = 1.35^25^. In order to meet the demand of the application market at a low cost, we combine the bidirectional structure with QCW fiber laser to achieve higher power and efficiency.

In this manuscript, a bidirectional output QCW fiber laser oscillator was constructed to achieve high power laser with good beam quality. Using a DCYDF with a core/cladding diameter of 25/400 μm, a near-single-mode QCW laser output of 2 × 4 kW is achieved with an optical-to-optical (O–O) efficiency of about 81% under the average pump power of 925 W. In order to increase the power, based on the impact of laser diode (LD) water-cooling temperature on pump absorption, we theoretically study the effect of fiber length on stimulated Raman scattering (SRS) in QCW fiber lasers. And then, by shortening the gain fiber length and reducing the SRS strength, the peak output power is further increased to 2 × 4.5 kW, corresponding to the O–O efficiency of about 79%.

## Experimental setup

**Figure 1:**
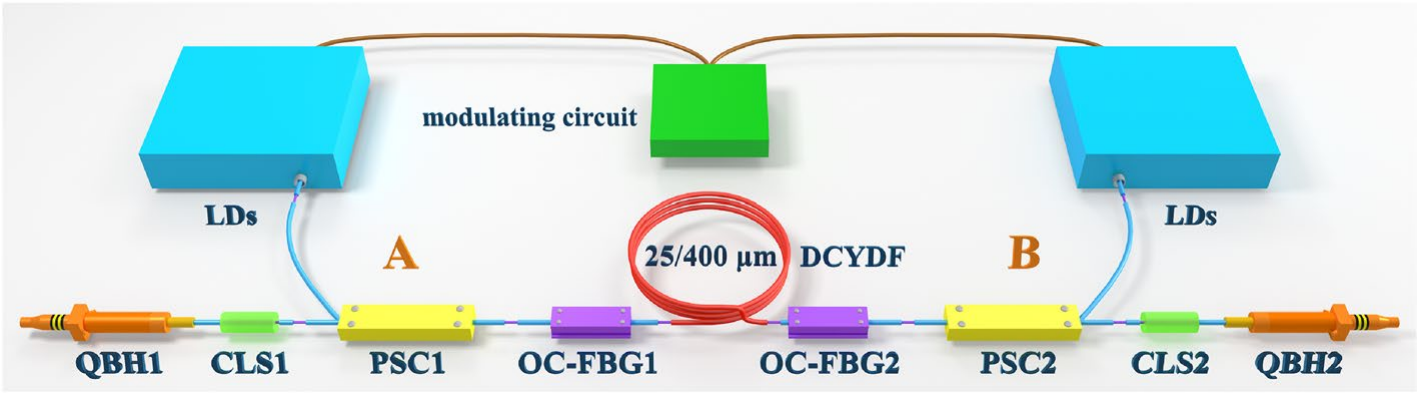
Schematic diagram of QCW fiber laser with bidirectional output.

The experimental setup of the bidirectional output QCW fiber laser is shown in Fig. 1. The fiber oscillator is composed of a gain fiber with a length of 25 m and two output-coupled fiber Bragg Gratings (OC-FBGs). The gain fiber used is DCYDF with a core/cladding diameter of 25/400 μm, the cladding absorption coefficient is 0.56 dB/m at 915 nm, and the core NA is 0.06. The center wavelength of OC-FBG1 is 1079.96 nm, the reflectivity is 5.3%, and the 3-dB bandwidth is 1.05 nm. The center wavelength of OC-FBG2 is 1079.95 nm, the reflectivity is 5.2%, and the 3-dB bandwidth is 1.04 nm. The pump power is injected into the oscillator through the pump and signal combiners (PSCs). Two (18 + 1) × 1 PSCs are connected to 17 LDs with a wavelength-stabilized 976 nm to provide the pump source, and the remaining pump arm with around 0° angle is used to measure the leaked light. The QCW laser is generated by directly modulating the driving current of LDs using the modulating circuit. In our experiment, the modulation frequency is set as 1 kHz, and the duty cycle is set as 10%. The signal light is output from the OC-FBG1 and OC-FBG2, the leaked signal light and residual pump light are filtered by the cladding light stripper (CLS1 and CLS2) and finally output from the quartz block head (QBH1 and QBH2) at both ends. We call the end where QBH1 is located the A end and the other end the B.

The laser output by QBH enters the test system through a collimator (CO), and the power is measured by a power meter (PM). An optical spectrum analyzer (OSA) with a wavelength range of 600–1700 nm was used to detect the scattered light from the PM target surface and analyze the output laser spectrum. The photodetector (PD) is connected to an oscilloscope to measure the time-domain characteristics of the output laser, which has an actual measurement bandwidth of 1 GHz. A band-pass optical filter (OF) is placed in front of the beam quality analyzer (BQA) with an operating wavelength range of 266–1100 nm to filter light other than the signal light band to measure beam quality. The center wavelength of the OF is 1075 nm and bandwidth is 50 nm.

## Results and discussion

### Effect of LD water-cooling temperature on pump absorption

In Ref.^28^, wavelength-stabilized LDs of 981 nm were used to achieve a bidirectional QCW laser output with a total peak power of 3 kW for the first time, and the peak power at both ends was 1218 W and 2220 W, respectively. However, the absorption cross section at 981 nm is small. This results in a relatively low O–O efficiency of 60%, limiting the output power further scaling.

**Figure 2:**
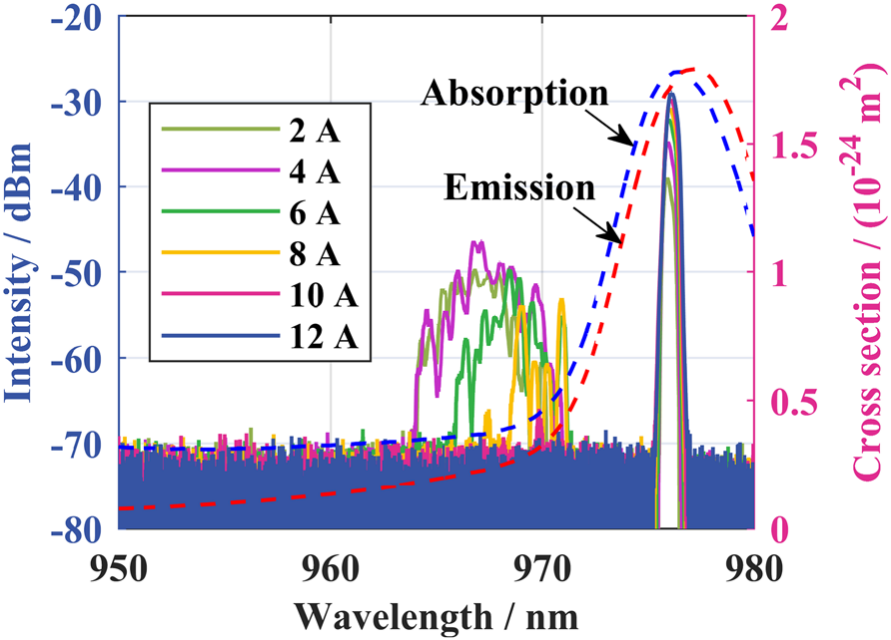
Different CW currents drive the LD output spectrum with corresponding absorption and emission cross section curves of YDF.

Since the absorption/emission cross-section curve of YDF reaches its peak at 976 nm, to improve the O–O efficiency and output power of the bidirectional output QCW fiber laser, wavelength-stabilized 976 nm LDs were used for pumping. Firstly, LDs were tested in continuous pumping status. The spectrum under different currents is shown in Fig. 2. The spectrum mainly comprises the prominent peak of 976 nm and the sub-peak of 963–973 nm. With the current increase, the waste heat accumulation in the LDs increases, resulting in the frequency shift from the sub-peak to the prominent peak and the power proportion gradually decreases. When the current exceeds 10 A, the sub-peak disappears and the energy is concentrated near 976 nm.

However, under QCW modulation, the laser output time is shorter and the heat accumulation in LDs is less, resulting in higher sub-peak energy. As shown in Fig. 2, the absorption cross-section at 963–973 nm is 3.39 × 10^–25^ to 1.04 × 10^−24^ m^2^, while the absorption cross-section at 976 nm is 1.77 × 10^–24^ m^2^, and the highest absorption cross-section at the sub-peak band is only 59% of that at 976 nm. Therefore, the absorption of the QCW pump will be significantly lower than that at the CW pump.

**Figure 3:**
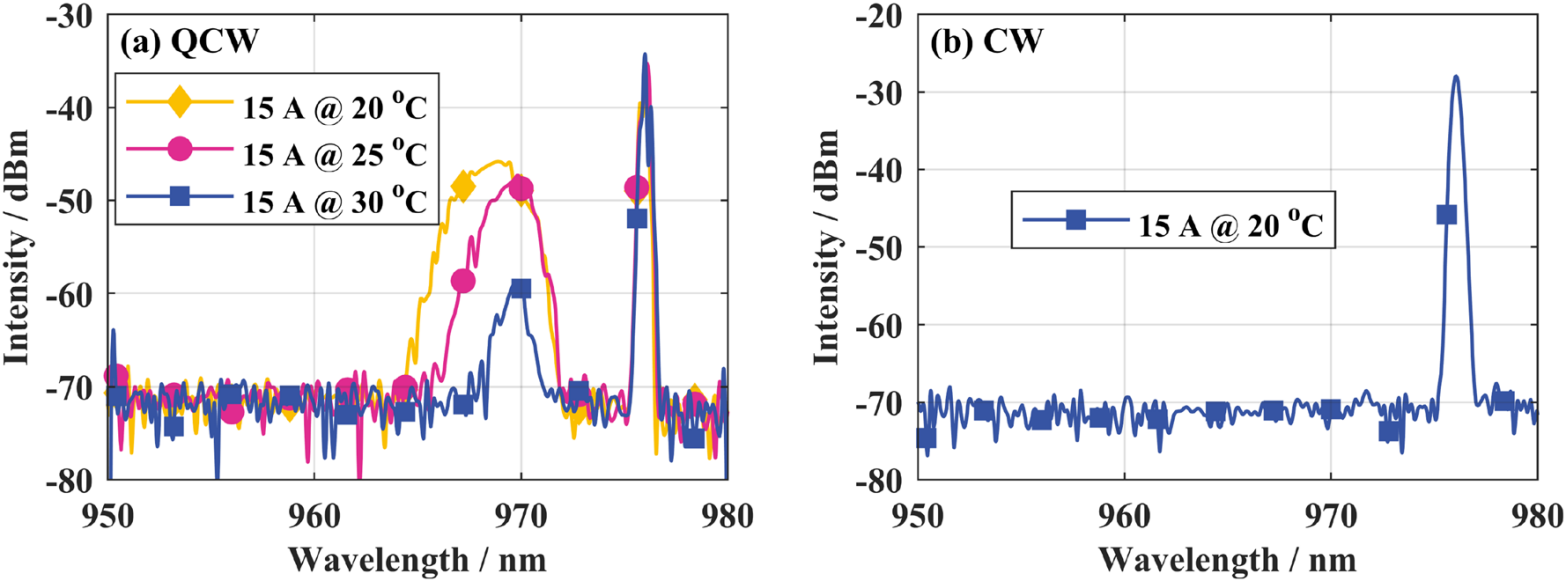
LD output spectrum: (**a**) driven by QCW current; (**b**) driven by CW current Optical fiber absorption coefficient *β* (dB/m) meets^29^:

The LD output spectrum at different temperatures in QCW status was measured to improve the pump spectrum shape and increase the pump absorption. As shown in Fig. 3, the water-cooling temperature of the pumped LD is increased from 20 to 30 °C. Driven by a current with a repetition rate of 1 kHz and a duty cycle of 10%, the sub-peak center wavelength of the pumped LD spectrum shifts to the long-wave direction by about 1 nm, the relative intensity decreases by about 13 dB, and the power ratio decreases from 66.7 to 3.9%. At the same time, the output average power of the single LD remains the same at about 75 W.

1 $$\beta = - \frac{10}{L}{\text{log}}\left( {\frac{{P_{out} }}{{P_{in} }}} \right)$$

Let *β*_*eff*_ be the equivalent absorption coefficient and *β*_*eff*_*L* is the total absorption of the pump light after passing through the gain fiber with length *L*. Then we can see from the above formula:

2 $$\int {P_{in} \left( \lambda \right) \times 10^{{ - \frac{\beta (\lambda )L}{{10}}}} } d\lambda = \int {P_{in} \left( \lambda \right)} d\lambda \times 10^{{ - \frac{{\beta_{eff} L}}{10}}}$$

*β*(*λ*) is the absorption coefficient at different wavelengths.

By calculating the absorption of the DCYDF with a length of 25 m and an absorption coefficient of 0.56 dB/m at 915 nm, when the water-cooling temperature is 20 °C, the total absorption is 10.46 dB, and the corresponding equivalent absorption coefficient is 0.42 dB/m. When the water-cooling temperature is 30 °C, the complete absorption is 20.39 dB, and the corresponding equivalent absorption coefficient is 0.82 dB/m. Appropriately increasing the water-cooling temperature helps to absorb the pump light, thereby improving the O–O efficiency.

### Experiment results of 25 m DCYDF

**Figure 4:**
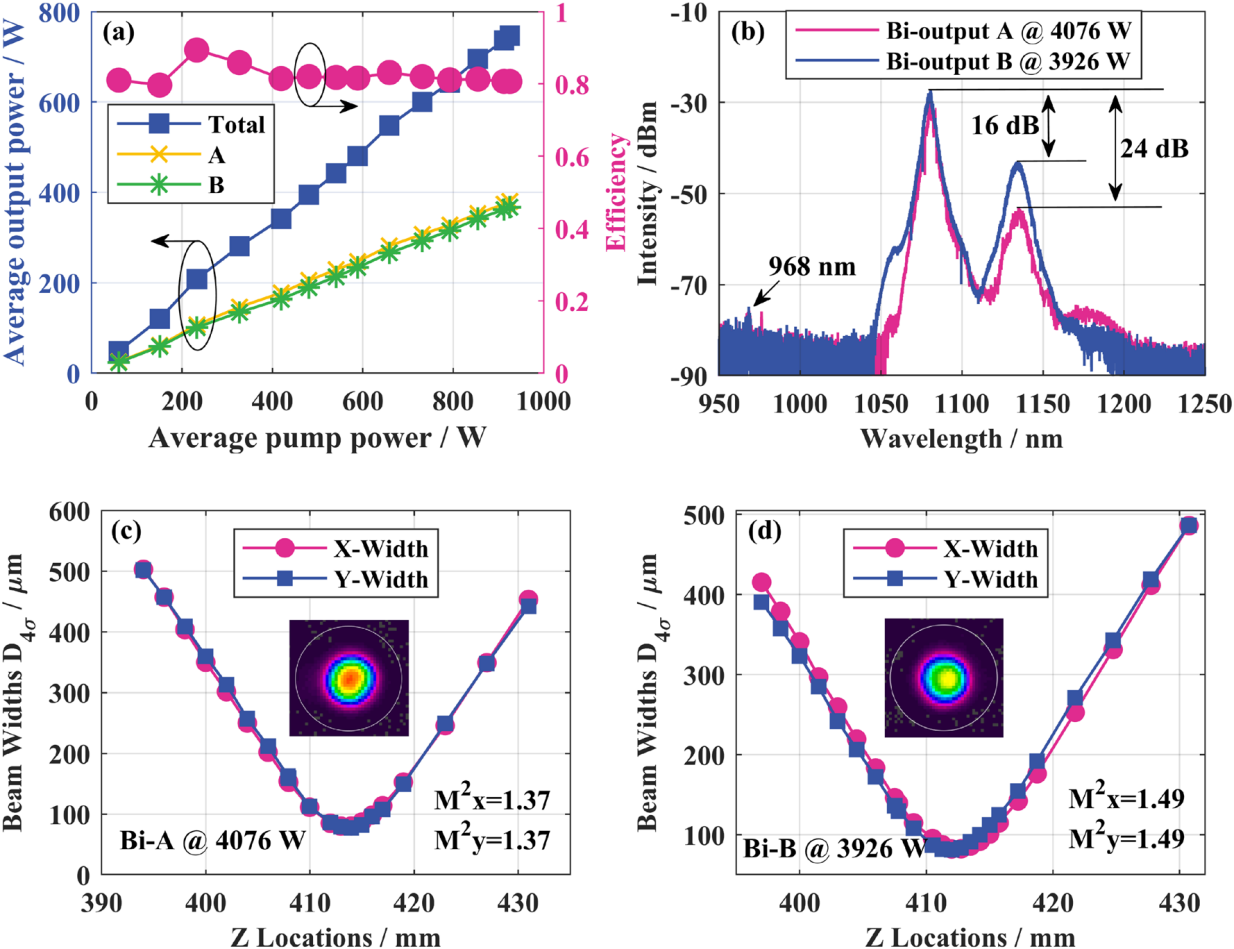
Experimental results of bidirectional output QCW fiber laser with peak power of 8002 W: (**a**) variation curves of the output power and O–O efficiency with the pump power; (**b**) output spectrum; (**c**) A-end beam quality and profile at maximum output power; (**d**) B-end beam quality and profile at maximum output power.

Based on the above experimental structure, set the DCYDF to 25 m firstly to ensure pump absorption. In order to improve the pump spectrum form and make the test devices work at a suitable temperature, the water-cooling temperature of the LDs was set to 30 °C, and the water-cooling temperature of the other devices were set to 20 °C. In the experiment, we first measured the average output power of the QCW laser. As shown in Fig. 4a, there is a good linear relationship between the output power and pump power. When the average pump power is above 400 W, the O–O efficiency remains at about 81%, and the power ratio difference between A and B ends does not exceed 5%. When the average pump power is 925 W, the total average output power reaches 747 W. The corresponding peak power is 8002 W, of which the peak power at the A end is 4076 W and the peak power at the B end is 3926 W. Figure 4b shows the output spectrum when the two ends reach the maximum output power. The Raman suppression ratio at the A end is 24 dB, and at the B end is 16 dB. Residual pumping light was detected at 968 nm. As shown in Fig. 4c,d, at the highest output power, the beam quality factor *M*^2^ at end A is 1.37 and at end B is 1.49. The maximum beam quality degradation at both ends is 0.06 during the experiment.

### Theory simulation and cavity optimization

In order to increase the output power, we need to optimize the laser in theory. Here we first give the theory of the simplified bidirectional output QCW laser, and then optimize the laser through simulation. Pump power, transverse mode instability (TMI), and nonlinear effects represented by SRS are the main factors limiting high-power fiber lasers’ power scaling under LD pumping^30^. At the same peak power, the average power of QCW fiber laser is lower compared to CW laser. In fact, the maximum average output power reached in the above experiment is 747 W, and there is still 481 W gap to rise with the TMI threshold of 1228 W under the CW pump. As a result, there is less heat accumulation, reducing the likelihood of thermal refractive index grating-induced TMI. Additionally, recent studies have shown that small-duty cycle pump modulation on the order of kHz can inhibit TMI^31,32^. Therefore, controlling SRS intensity is particularly important for high-power QCW fiber lasers and TMI is not considered in our simulation.

The peak power scaling of the above experiments is also limited by SRS. In order to suppress SRS, the effect of fiber length on SRS intensity was studied based on the theoretical model of bidirectional output QCW fiber laser, using the modified steady-state rate equation^19,24,28,29,33,34^. The main simulation equations are (3)–(5).

3 $$\begin{aligned} \pm \frac{{{\text{d}}P_{n}^{{{\text{s}} \pm }} \left( {\lambda_{n}^{{\text{s}}} ,z} \right)}}{{{\text{d}}z}} & = \Gamma_{{\text{s}}} \left[ {\sigma_{n}^{{{\text{es}}}} \left( {\lambda_{n}^{{\text{s}}} } \right)N_{2} \left( z \right) - \sigma_{n}^{{{\text{as}}}} \left( {\lambda_{n}^{{\text{s}}} } \right)N_{1} \left( z \right)} \right]P_{n}^{{{\text{s}} \pm }} \left( {\lambda_{n}^{{\text{s}}} ,z} \right) \\ & \quad + 2\sigma_{n}^{{{\text{es}}}} \left( {\lambda_{n}^{{\text{s}}} } \right)N_{2} \left( z \right)\frac{{hc^{2} }}{{\left( {\lambda_{n}^{s} } \right)^{3} }}\Delta \lambda - a_{n}^{{\text{s}}} \left( {\lambda_{n}^{s} } \right)P_{n}^{{{\text{s}} \pm }} \left( {\lambda_{n}^{{\text{s}}} ,z} \right) \\ & \quad + \Gamma_{n}^{{\text{s}}} \left( {\lambda_{n}^{{\text{s}}} } \right)P_{n}^{{{\text{s}} \pm }} \left( {\lambda_{n}^{{\text{s}}} ,z} \right)\mathop \sum \limits_{i = 1}^{N} \frac{1}{{A_{{{\text{core}}}}^{i,n} }}g_{{\text{R}}} \left( {\omega_{i} - \omega_{n} } \right)\left[ {P_{i}^{{{\text{s}} + }} \left( {\lambda_{i}^{{\text{s}}} ,z} \right) + P_{i}^{{{\text{s}} - }} \left( {\lambda_{i}^{{\text{s}}} ,z} \right)} \right] \\ \end{aligned}$$

4 $$\begin{aligned} \pm \frac{{{\text{d}}P_{m}^{{{\text{p}} \pm }} \left( {\lambda_{m}^{{\text{p}}} ,z} \right)}}{{{\text{d}}z}} & = \Gamma_{{\text{p}}} \left[ {\sigma_{m}^{{{\text{ep}}}} \left( {\lambda_{m}^{{\text{p}}} } \right)N_{2} \left( z \right) - \sigma_{m}^{{{\text{ap}}}} \left( {\lambda_{m}^{{\text{p}}} } \right)N_{1} \left( z \right)} \right]P_{m}^{{{\text{p}} \pm }} \left( {\lambda_{m}^{{\text{p}}} ,z} \right) \\ \quad - a_{m}^{{\text{p}}} \left( {\lambda_{m}^{{\text{p}}} } \right)P_{m}^{{{\text{p}} \pm }} \left( {\lambda_{m}^{{\text{p}}} ,z} \right) \\ \end{aligned}$$

5 $$\begin{aligned} \frac{{\partial N_{2} \left( {z,t} \right)}}{\partial t} & = \frac{{\Gamma_{{\text{p}}} \lambda_{p} }}{{A_{{{\text{core}}}} hc}}[P_{{\text{p}}}^{ + } \left( z \right) + P_{{\text{p}}}^{ - } \left( z \right)][\sigma_{{{\text{ap}}}} N_{1} - \sigma_{{{\text{ep}}}} N_{2} ] \\ & \quad + \frac{{\Gamma_{{\text{s}}} \lambda_{s} }}{{A_{{{\text{core}}}} hc}}[P_{{\text{s}}}^{ + } \left( z \right) + P_{{\text{s}}}^{ - } \left( z \right)][\sigma_{{{\text{as}}}} N_{1} - \sigma_{{{\text{es}}}} N_{2} ] \\ & \quad - \frac{{N_{2} }}{\tau } \\ \end{aligned}$$

where *P* is the power of laser; *Г* is the overlap factor; *N*_1_ is the ground state particle number density; *N*_2_ is the excited state particle number density; *σ*^*a*^ is the absorption cross section; *σ*^*e*^ is the emission cross section; *λ* is the wavelength; *Δλ* is the gain spectral bandwidth; *g*_*R*_ is the Raman gain; *ω* is the angular frequency; *α* is the attenuation coefficient; *A*_*core*_ is the core area; *τ* is the lifetime of excited particles; h is the Planck constant; *c* is the light velocity in vacuum.

**Figure 5:**
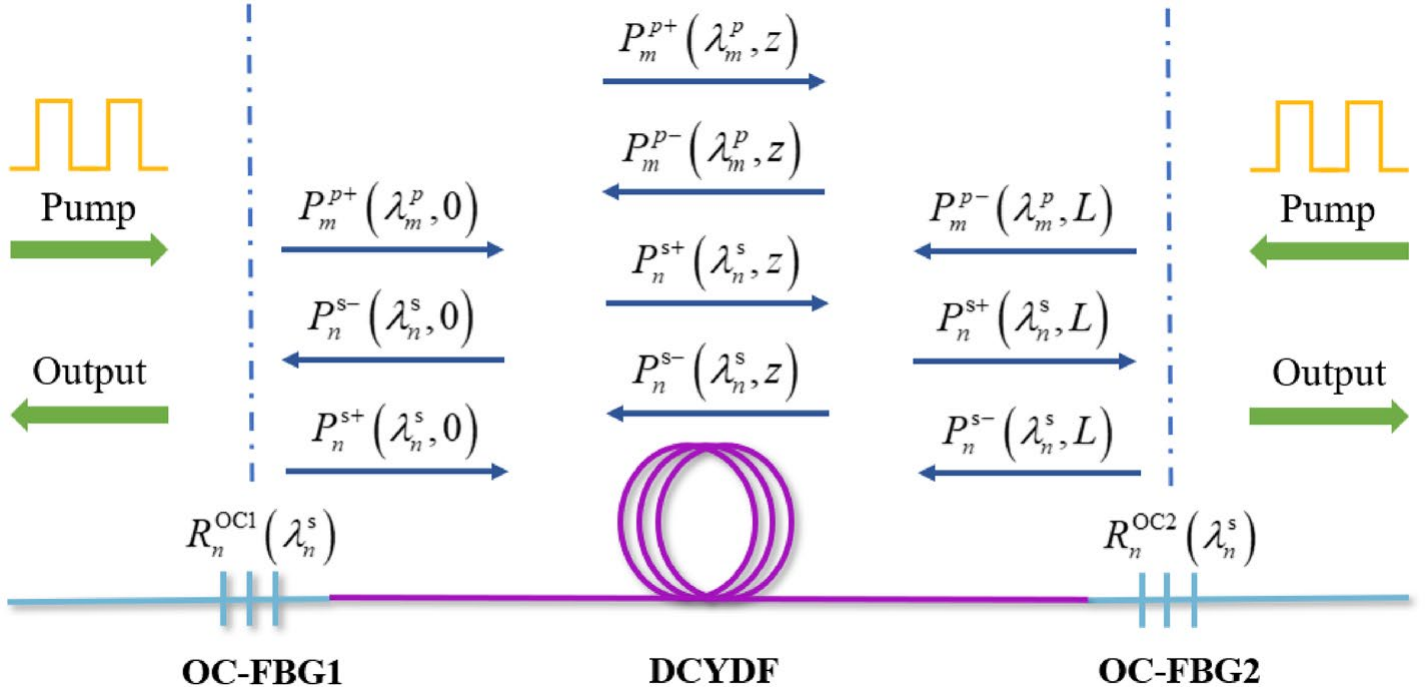
Schematic diagram of the theoretical model of bidirectional output QCW fiber laser.

The essential difference between the bidirectional output structure and the unidirectional structure is that high reflectivity fiber Bragg grating (HR-FBG) is replaced by output coupler fiber Bragg grating (OC-FBG), and the corresponding boundary conditions are also changed, as shown in Fig. 5. Due to the transmission and reflection of the grating, the boundary conditions of the signal light are shown in Eqs. (6). The grating is considered to have only a transmission effect on the pump light, as shown in formula (7). The parameters used in the simulation are shown in Table 1.

6 $$\left\{ \begin{aligned} & P_{n}^{{{\text{s}} + }} \left( {\lambda_{n}^{{\text{s}}} ,0} \right) = P_{n}^{{{\text{s}} - }} \left( {\lambda_{n}^{{\text{s}}} ,0} \right)R_{n}^{{{\text{OC}}1}} \left( {\lambda_{n}^{{\text{s}}} } \right) + P_{noise} \\ & P_{n}^{{{\text{s}} - }} \left( {\lambda_{n}^{{\text{s}}} ,L} \right) = P_{n}^{{{\text{s}} + }} \left( {\lambda_{n}^{{\text{s}}} ,L} \right)R_{n}^{{{\text{OC}}2}} \left( {\lambda_{n}^{{\text{s}}} } \right) + P_{noise} \\ & P_{n}^{{{\text{s}} - }} \left( {\lambda_{n}^{{\text{s}}} ,0_{out} } \right) = P_{n}^{{{\text{s}} - }} \left( {\lambda_{n}^{{\text{s}}} ,0} \right)\left[ {1 - R_{n}^{{{\text{OC}}1}} \left( {\lambda_{n}^{{\text{s}}} } \right)} \right] \\ & P_{n}^{{{\text{s}} + }} \left( {\lambda_{n}^{{\text{s}}} ,L_{out} } \right) = P_{n}^{{{\text{s}} + }} \left( {\lambda_{n}^{{\text{s}}} ,L} \right)\left[ {1 - R_{n}^{{{\text{OC}}2}} \left( {\lambda_{n}^{{\text{s}}} } \right)} \right] \\ \end{aligned} \right.$$

7 $$\left\{ \begin{aligned} & P_{m}^{p + } \left( {\lambda_{m}^{p} ,0} \right) = P_{m}^{p0 + } \left( {\lambda_{m}^{p} } \right) \\ & P_{m}^{p - } \left( {\lambda_{m}^{p} ,L} \right) = P_{m}^{p0 - } \left( {\lambda_{m}^{p} } \right) \\ \end{aligned} \right.$$

**Table 1:** Main parameters and values in theoretical simulation.

Parameter	Value	Parameter	Value
Core/Cladding diameter	25/400 μm	Core numerical aperture (NA)	0.06
Cladding absorption coefficient	1.74 dB/m @976 nm	Core/Cladding attenuation	5.6/1.7 dB/km
Pump wavelength	976 nm	Signal wavelength	1080 nm
Pump power overlap factor	0.0025	Signal power overlap factor	1
Average pump power at both ends	500 W	Repetition frequency	1 kHz
Duty cycle	10%	Center wavelength of OC-FBG	1080 nm
Reflectivity of OC-FBG	5%	3-dB bandwidth of OC-FBG	1 nm

**Figure 6:**
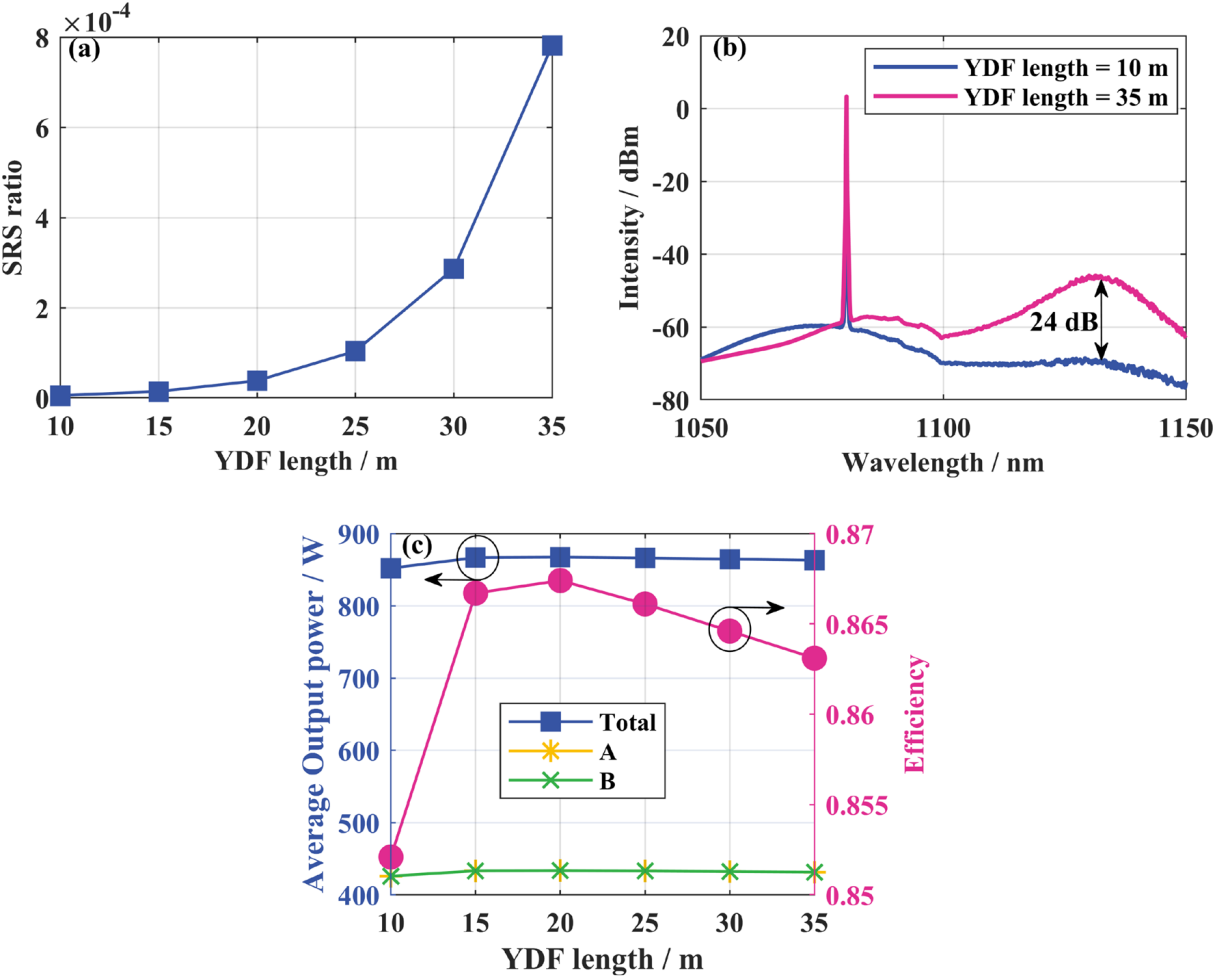
(**a**) the Raman power ratio varies with the length of the gain fiber; (**b**) Output spectrum at different fiber lengths; (**c**) the variation of output power and O–O efficiency with the length of the gain fiber.

Through simulation analysis, the qualitative conclusions of SRS intensity and O–O efficiency with fiber length is obtained. The O–O efficiency in simulation is obviously higher than that in experiment because the device loss and high order mode loss are not considered. Figure 6a,b shows that when the fiber length increases from 10 to 35 m, the SRS strength increases by about 24 dB and the Raman power ratio increases from 5.85 × 10^–6^ to 7.82 × 10^–4^. Therefore, reducing the gain fiber length helps reduce the SRS intensity. However, the length of the gain fiber is too short, which will lead to insufficient pump absorption and low O–O efficiency. As shown in Fig. 6c, when the gain fiber length is less than 20 m, the pump absorption is inadequate, and the power and efficiency increase rapidly with the increase of the gain fiber length. When the fiber length exceeds 20 m, the gain fiber length increases, and the power and efficiency gradually decrease due to loss. Therefore, it is essential to consider the O–O efficiency and SRS intensity comprehensively when designing the gain fiber length.

### Experiment results of 20 m DCYDF

**Figure 7:**
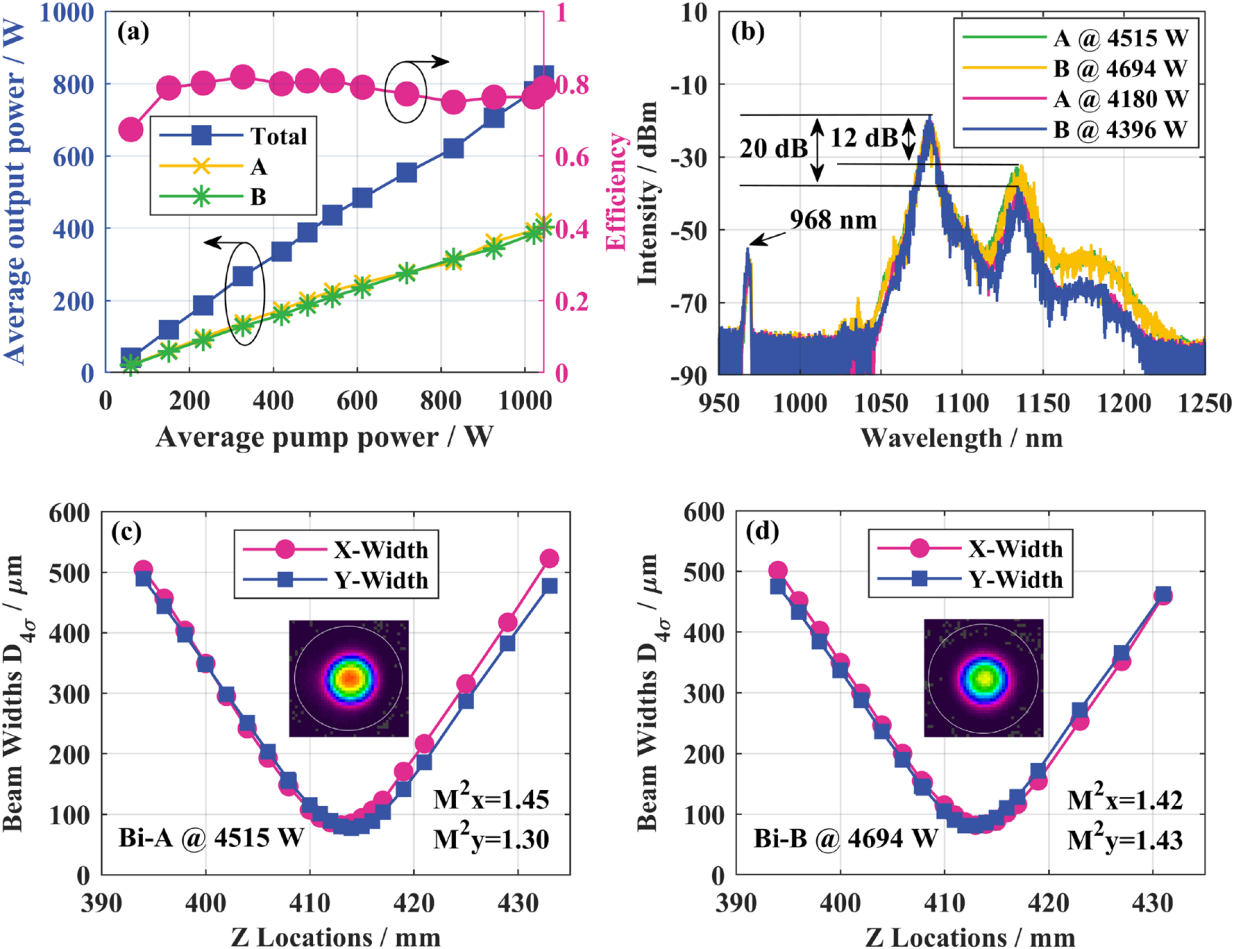
Experimental results of bidirectional output QCW fiber laser with peak power of 9209 W: (**a**) variation curves of the output power and O–O efficiency with the pump power; (**b**) output spectrum; (**c**) A-end beam quality and profile at maximum output power; (**d**) B-end beam quality and profile at maximum output power.

According to the simulation results, the SRS intensity can be reduced while the efficiency can hold at a higher one by shortening the fiber length. To get a higher power scaling, we reduced the gain fiber length from 25 to 20 m. When the water-cooling temperature of the LDs is 30°, the total pump absorption is 18.38 dB at this length, corresponding to the equivalent absorption coefficient of 0.92 dB/m. In this case, the experimental results are shown in Fig. 7. In Fig. 7a, pump and output power still maintain a good linear relationship. Possibly because of the temperature fluctuation of LDs, the O–O efficiency fluctuates slightly with increasing pump power. But except for the point where the average pump power is 60 W, it is maintained at more than 75%. When the average pump power is 1045 W, the O–O efficiency is 79%, and the average output power of A and B reaches the maximum of 419 W and 404 W, respectively. The total peak output power of the laser currently is 9209 W, and the corresponding peak power at both ends of A and B are 4515 W and 4694 W, respectively. The output spectrum is shown in Fig. 7b, and the Raman suppression ratio at the maximum output power is 12 dB. When the total peak power is 8576 W, the Raman suppression ratio is 20 dB. Compared with 25 m gain fiber length, the peak power of the same Raman suppression ratio (24 dB @4076 W at end A, 16 dB @3926 W at end B) increased by 574 W. This means that shortening the gain fiber length helps to reduce the SRS and can scale up the output power. As shown in Fig. 7c,d, under the maximum output power, the beam quality factor at the A end is *M*^2^_*x*_ = 1.45 and *M*^2^_*y*_ = 1.30; The beam quality factor at the B end is *M*^2^_*x*_ = 1.42 and *M*^2^_*y*_ = 1.43.

**Figure 8:**
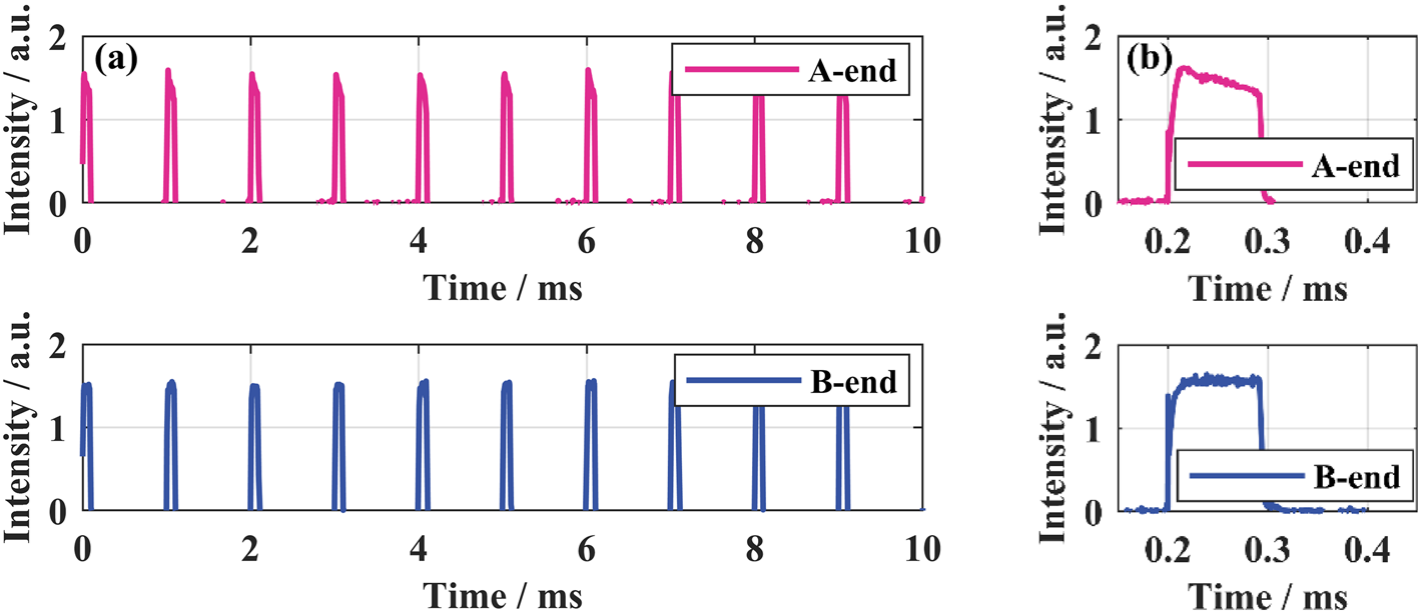
The time-domain pulse profile at a peak power of 9209 W: (**a**) pulse train; (**b**) single pulse.

The output pulse amplitude in the time domain was measured and shown in Fig. 8. The actual output repetition frequency was 1 kHz, and the pulse width was about 94.5 μs. The output pulses at the highest peak power in both A and B ends were stable without TMI, indicating a considerable long-term and short-term output power and beam quality stability.

## Conclusion

Based on the theoretical model of bidirectional output QCW fiber laser oscillator, it is verified that the SRS intensity increases with the increase of gain fiber length. Pumped with 976 nm LDs and based on fiber with a core/cladding diameter of 25/400 μm, QCW laser output with a total peak power of 8 kW is achieved, with peak power of 4076 W and 3926 W at two ends, corresponding beam quality factors of *M*^2^ = 1.37 and *M*^2^ = 1.49, respectively. At the same time, the LD water-cooling temperature was increased to 30 °C during the experiment, thereby improving the O–O efficiency to 81%. Therefore, to reduce the SRS intensity and increase the power scaling, we reduced the gain fiber length from 25 to 20 m, obtaining a QCW laser output with a peak power of 9 kW (4515 W at the A end and 4694 W at the B end) and a Raman suppression ratio of 12 dB, where the beam quality factor at end A is *M*^2^_*x*_ = 1.45 and *M*^*2*^_*y*_ = 1.30 and it at the B end is *M*^2^_*x*_ = 1.42 and *M*^2^_*y*_ = 1.43. The power increase of the QCW fiber laser is limited by SRS, and the subsequent use of fibers with larger absorption coefficients or special fibers is expected to achieve near-single-mode output with higher power scaling.

## Data Availability

The datasets used and/or analyzed during the current study available from the corresponding author upon reasonable request.
